# Artificial Leaf for Solar‐Driven Ammonia Conversion at Milligram‐Scale Using Triple Junction III‐V Photoelectrode

**DOI:** 10.1002/advs.202205808

**Published:** 2023-03-22

**Authors:** Hao Huang, Dharmaraj Periyanagounder, Cailing Chen, Zhongxiao Li, Qiong Lei, Yu Han, Kuo‐Wei Huang, Jr‐Hau He

**Affiliations:** ^1^ KAUST Catalysis Center King Abdullah University of Science and Technology Thuwal 23955–6900 Saudi Arabia; ^2^ Division of Physical Sciences and Engineering King Abdullah University of Science and Technology Thuwal 23955–6900 Saudi Arabia; ^3^ Advanced Membrances and Porous Materials Center King Abdullah University of Science and Technology Thuwal 23955–6900 Saudi Arabia; ^4^ Department of Materials Science and Engineering City University of Hong Kong Kowloon Hong Kong SAR 999077 P. R. China

**Keywords:** artificial leaf, large‐scale, nitrogen fixation, solar‐to‐ammonia conversion, triple‐junction cell

## Abstract

Developing a green and energy‐saving alternative to the traditional Haber‐Bosch process for converting nitrogen into ammonia is urgently needed. Imitating from biological nitrogen fixation and photosynthesis processes, this work develops a monolithic artificial leaf based on triple junction (3J) InGaP/GaAs/Ge cell for solar‐driven ammonia conversion under ambient conditions. A gold layer serves as the catalytic site for nitrogen fixation with photogenerated electrons. The Au/Ti/3J InGaP/GaAs/Ge photoelectrochemical (PEC) device achieves high ammonia production rates and Faradaic efficiencies in a two‐electrode system without applying external potential. For example, at 0.2 sunlight intensity, the solar‐to‐ammonia (STA) conversion efficiency reaches 1.11% and the corresponding Faradaic efficiency is up to 28.9%. By integrating a Ni foil on the anode side for the oxygen evolution reaction (OER), the monolithic artificial leaf exhibits an ammonia production rate of 8.5 µg cm^−2^ h at 1.5 sunlight intensity. Additionally, a 3 × 3 cm unassisted wireless PEC device is fabricated that produces 1.0039 mg of ammonia in the 36‐h durability test. Thus, the new artificial leaf can successfully and directly convert solar energy into chemical energy and generate useful products in an environmentally friendly approach.

## Graphical abstract

A monolithic artificial leaf device is developed for solar‐driven nitrogen fixation, achieving the highest solar‐to‐ammonia conversion efficiency of 1.11% and ammonia production at mg‐scale.

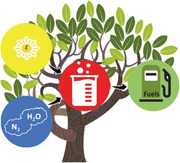

## 1 Introduction

Ammonia is a widely used intermediate in chemical industry and agriculture. Fixing nitrogen gas into ammonia from atomsphere is becoming increasing improtant for the human society.^[^ ^1^, ^2^ ^]^ At present, the industrial Haber–Bosch (HB) process is mainly used to synthesize N‐containing products at the hundred‐million ton‐scale. However, the process requires harsh reaction conditions (650–750 K and 50–200 bar atm) and consumes 1–3% of the global energy, resulting in the release of a large amount of CO_2_ gas and causing serious environmental problems.^[^ ^3^ ^]^ Therefore, solar‐driven ammonia synthesis from N_2_ and water under ambient conditions is emerging as an effective alternative to the HB process.^[^ ^4^, ^5^ ^]^

Considerable efforts have been made to achieve the solar energy‐driven nitrogen reduction reaction (NRR) using photochemcial and photoelectrochemical (PEC) processes.^[^ ^6^, ^7^, ^8^, ^9^, ^10^, ^11^ ^]^ Compared to typical photochemical systems, PEC systems exhibit high efficiency for solar‐to‐chemical energy conversion, with broad light harvesting ability, facile charge carrier transfer processed, and rational catalyst integration ability.^[^ ^9^, ^10^, ^11^ ^]^ Thus far, Si, TiO_2_, and SrTiO_3_ have been primarily used as photoelectrodes to harvest solar energy.^[^ ^12^, ^13^, ^14^ ^]^ Light utilization was limited to UV and partial visible wavelength and an external bias potential was required to drive the overall redox reactions. Aiming to simulate natural photosynthesis and develop energy‐efficient PEC systems, artificial leaf devices that function monolithically and spontaneously under light illumination without any bias potentials and additional attachments have received considerable attention in water splitting and CO_2_ reduction reactions.^[^ ^15^, ^16^, ^17^, ^18^, ^19^, ^20^ ^]^ However, spontaneous nitrogen reduction requires a potential of 0.55 V versus normal hydrogen electrode to meet the thermodynamic requirements.^[^ ^21^, ^22^ ^]^ Considering the energy barrier for N_2_ activation and the overpotential of the oxygen evolution reaction (OER), the minimal photovoltage to drive the redox reactions should be higher than 1.80 V. However, none of the single photoelectrodes can produce such a high photovoltage. Accordingly, most PEC NRR works are based on half‐cells, which only simulate a part of the PEC system and not the overall solar‐driven nitrogen reduction process.^[^ ^7^, ^10^, ^23^, ^24^ ^]^ Furthermore, the half‐cells developed thus far cannot be easily connected in series, rendering the ongoing efforts in spontaneous solar‐driven ammonia production only a proof‐of‐concept. To overcome this limitation, tandem PEC cells, wherein p‐n junctions with different bandgaps are stacked to generate sufficient photovoltages, are expected for the large‐scale, highly efficient, and unassisted solar‐driven ammonia production in a distributed manner.

The effective fixation of nitrogen into ammonia using solar energy requires highly active catalysts to activate the inert N_2_ molecules (940.05 kJ mol^−1^) and suppress the competitive hydrogen evolution reaction (HER). A variety of Au‐based catalysts have been utilized for solar‐driven CO_2_ reduction reaction and NRR.^[^ ^7^, ^13^, ^14^, ^25^, ^26^, ^27^ ^]^ Au catalysts can be easily deposited through atomic layer deposition and thermal sputtering for realizing PEC reactions. Although Au catalysts have shown the highest activity and efficiency in unassisted PEC NRR systems thus far, the production rates at very low current densities limit the solar‐driven ammonia conversion at the µg scale or lower.^[^ ^12^, ^13^, ^14^ ^]^ The ammonia yields were lower than 1.33 µg cm^−2^ h, which is much lower than the requirement for practical applications.^[^ ^12^ ^]^ Further increase in the current density results in the dominance of HER and decreases the overall nitrogen reduction efficiency.^[^ ^10^ ^]^ This renders the fabrication of PEC NRR systems with both high conversion efficiency and high ammonia yield difficult.

In this study, we have developed an artificial leaf using Au/Ti/triple junction (3J) InGaP/GaAs/Ge/Ni to produce 2.37 V for wireless solar‐to‐ammonia (STA) conversion under 1 sun AM 1.5G illumination. The Au film with a 10 nm thick Ti transition layer adhered to the 3J InGaP/GaAs/Ge cell surface firmly and was translucent under light illumination, exhibiting superior stability. The highest ammonia conversion rate (12.4 µg cm^−2^ h) was achieved at 1.5 sunlight intensity and the highest STA efficiency (1.11%) was achieved at 0.2 sunlight intensity in the unassisted two‐electrode PEC system. The high ammonia conversion rate and STA efficiency were attributed to the appropriate photoinduced charge carrier densities associated with the highly active NRR catalyst. A fabricated 3 × 3 cm 3J‐based wireless artificial leaf produced 1.0039 mg of ammonia in 36‐h under 1.5 sun illumination, demonstrating the practical application of this device. This mg‐scale production of ammonia achieved in this work sets a roadmap for navigating the pathway toward the establishment of an unassisted wireless artificial leaf device for large‐scale solar‐to‐chemical energy conversion technologies.

## 2 Results and Discussion

**Figure 1:**
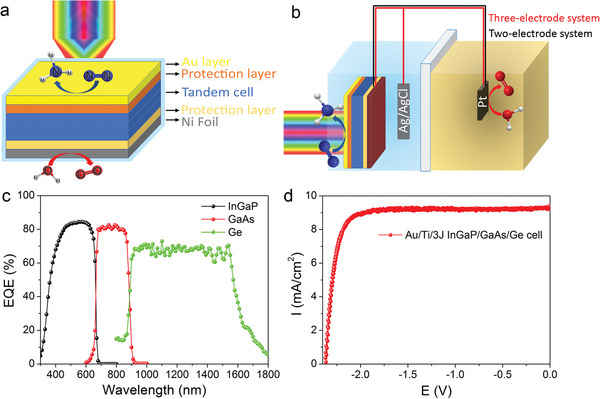
Illustration of the three photoelectrochemical (PEC) systems and the photovoltaic (PV) performance of the Au/Ti/3J InGaP/GaAs/Ge cell. a) Schematic illustration of the artificial leaf for nitrogen reduction using a 3J InGaP/GaAs/Ge cell with Au catalyst for the nitrogen reduction reaction (NRR) and Ni foil for the oxygen evolution reaction (OER). b) Schematic illustration of the three‐electrode PEC system (red circuit) and the unassisted two‐electrode PEC system (black circuit) using a 3J InGaP/GaAs/Ge cell with the Au catalyst as the photoanode for the NRR, Ag/AgCl as the reference electrode, and Pt as the counter electrode for the OER. c) External quantum efficiency (EQE) measurements of the 3J InGaP/GaAs/Ge cell. d) *J–V* characteristics of the Au/Ti/3J InGaP/GaAs/Ge cell under ambient conditions under 1 sun AM 1.5G illumination.

An artificial leaf is a new and succinct device that can convert solar energy into chemical energy. **Figure** **1**a presents the schematic of the artificial leaf for the green ammonia production using the Au/Ti/3J InGaP/GaAs/Ge cell/Ni foil PEC device. For comparison, the traditional three‐electrode PEC system (red circuit with Ag/AgCl reference electrode) and the unassisted two‐electrode PEC system (black circuit) using the Au/Ti/3J InGaP/GaAs/Ge cell as the photoanode are shown in Figure 1b. Here, the thin and transparent Au layer served as the NRR catalyst, and the Ni foil acted as the OER catalyst. A 10 nm thick Ti layer served as a protective layer and enhanced the electrons transfer between Au and InGaP. Owing to the cascade band structure of the 3J InGaP/GaAs/Ge cell, photoelectrons moved to Au/Ti surfaces for the NRR while the holes transferred to the Ni side for the OER under light illumination. The optical bandgaps of the semiconductors determined the spectral range of the absorbed photons. The corresponding external quantum efficiency (EQE) of the 3J InGaP/GaAs/Ge cell is shown in Figure 1c. The InGaP junction mainly absorbed visible light (300–700 nm) with the highest EQE of 84.46% at 570 nm. The GaAs junction absorbed near infrared light in the range of 600–900 nm, whereas the Ge junction absorbed infrared light in the range of 800–1700 nm. Thus, the three junctions of the cell could efficiently absorb solar light over a broad range of wavelength (visible to infrared) (Figure S1, Supporting Information). As shown in Figure 1d, under 1 sun AM 1.5G illumination the Au/Ti/3J InGaP/GaAs/Ge cell exhibited a short circuit current density (*J*_sc_) of 9.26 mA cm^−2^ at 0 V and an open‐circuit voltage (*V*_oc_) of 2.37 V. The fill factor (FF) reached 83.18%, as apparent from the linear sweep voltammetry (LSV) curve. Considering the overpotentials of the OER (1.4–1.6 V versus RHE) and NRR (−0.2 to −0.6 V versus RHE), the photovoltaic (PV) performance of the Au/Ti/3J InGaP/GaAs/Ge cell was high enough to spontaneously drive the redox reactions under light illumination.

**Figure 2:**
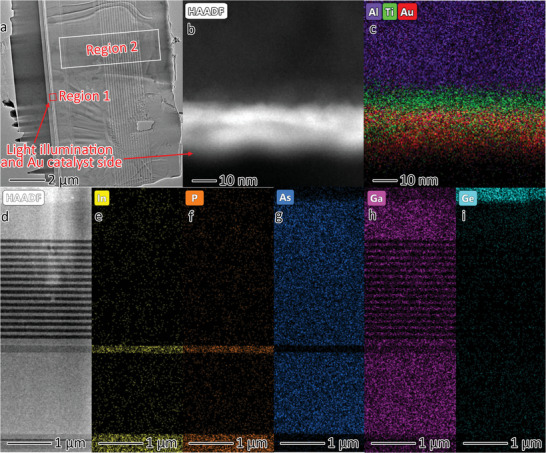
Characterization of the Au/Ti/3J InGaP/GaAs/Ge cell. a) Low‐resolution transmission electron microscopy (TEM) image of the Au/Ti/3J InGaP/GaAs/Ge cell. b) High‐angle annular dark field scanning transmission electron microscopy (HAADF–STEM) image of region 1 in (a), and c) the corresponding elemental mapping image. d) HAADF–STEM image of region 2 in (a), and e–i) the corresponding elemental mapping images.

To further reveal the composition and structure of the PEC device, transmission electron microscopy (TEM) and high‐angle annular dark field scanning transmission electron microscopy (HAADF‐STEM) with elemental distribution mapping of the cross section were conducted, as shown in **Figure** **2**a–i. The device was ≈6 µm thick, containing Au, Ti, and Al layers and InGaP, GaAs, and Ge semiconductors. Figure 2b,c shows the light illumination and Au catalyst in Region 1 of Figure 2a, respectively. The Au and Ti layers were approximately 20 and 10 nm thick, respectively. Figure 2d displays the HAADF‐STEM image of the 3J InGaP/GaAs/Ge cell in Region 2 of Figure 2a. Based on the elemental distribution in Figure 2e–i, the 500 nm thick InGaP junction (1.88 eV of the band gap) is displayed at the bottom. The GaAs junction (1.40 eV of the band gap) was approximately 5 µm thick and contained multiple quantum wells, in the middle of which there was an InGaP layer that acted as the tunnel junction. The 400 nm thick Ge junction (0.67 eV of the band gap) is displayed at the top of the Figure 2e–i.

**Figure 3:**
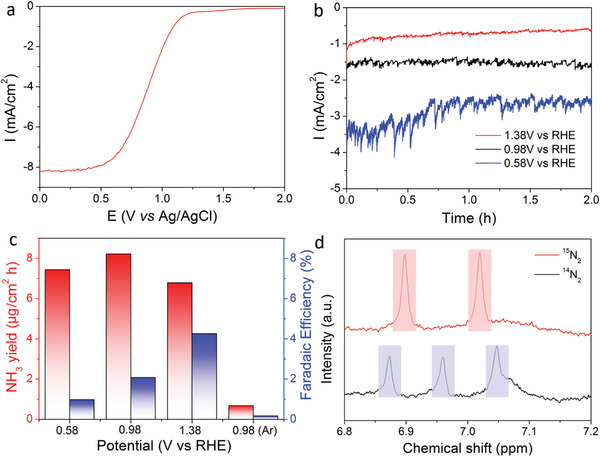
Nitrogen reduction reaction (NRR) performance of the Au/Ti/3J InGaP/GaAs/Ge photoelectrode in the three‐electrode photoelectrochemical (PEC) system. a) *J–V* curve of the photoelectrode in the three electrode PEC system. b) Chronoamperometry curve of the Au/Ti/3J InGaP/GaAs/Ge cell with various bias potential versus RHE. c) Corresponding ammonia production rate (left column) and Faradaic efficiency (right column) of the Au/Ti/3J InGaP/GaAs/Ge cell with various bias potential versus RHE. d) ^1^H NMR analysis of the electrolyte fed by ^15^N_2_ and ^14^N_2_ gases after 12 h of electrolysis at 0.98 V versus RHE. Reaction conditions: The Au/Ti/3J InGaP/GaAs/Ge cell was used as the photoanode in N_2_‐saturated 0.1 m NaOH electrolyte under 1 sunlight illumination with Ag/AgCl as the reference electrode and Pt as the cathode.

**Figure 4:**
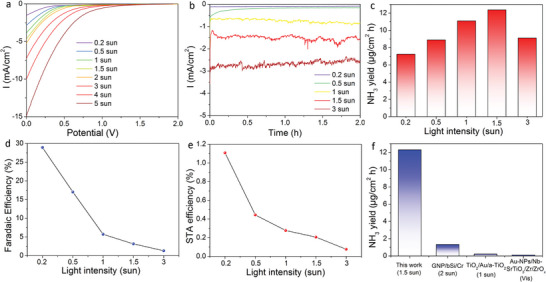
Nitrogen reduction reaction (NRR) performance of the Au/Ti/3J InGaP/GaAs/Ge photoelectrode in the two‐electrode wired photoelectrochemical (PEC) system. a) *J–V* curves and b) chronoamperometry curves of the photoelectrode in the two‐electrode wired PEC system under various light intensities. c) Corresponding ammonia production rate, d) Faradaic efficiency, and e) solar‐to‐ammonia (STA) efficiency of the two‐electrode wired PEC system under various light intensities. f) Comparison of the ammonia production rate of our two‐electrode wired PEC system with those of previously reported unassisted PEC NRR systems. Reaction conditions: The Au/Ti/3J InGaP/GaAs/Ge cell was used as the photoanode in N_2_‐saturated 0.1 m NaOH electrolyte and Pt as cathode.

We next evaluated the nitrogen fixation performance of the PEC device. First, we investigated the NRR activity in a three‐electrode system (Figure 1b, red circuit) using the Au/Ti/3J InGaP/GaAs/Ge cell as photoanode, which was exposed in N_2_ saturated 0.1 m NaOH electrolyte under 1 sun illumination. **Figure** **3**a shows the LSV curve of the Au/Ti/3J InGaP/GaAs/Ge photoanode. The maximum current density reached −8.2 mA cm^−2^ at 0 V versus Ag/AgCl. To exclude the potential contaminants from the feeding gases and electrolyte, we conducted the control experiments using gas chromatography (GC), UV–Vis spectroscopy, and nuclear magnetic resonance (NMR) spectroscopy (Figures S2–S4, Supporting Information). From Figure 2 (Supporting Information), only N_2_ gas was detected by GC without NO_x_ contaminant. Purified ^14^N_2_ gas and ^15^N_2_ gas were purged into deionized (DI) water and 0.1 m NaOH electrolyte for 15 min. The potential ammonia contaminants in the solutions were detected by UV–Vis absorption spectra and NMR. No ammonia was detected from ^14^N_2_ gas, ^15^N_2_ gas, and NaOH electrolyte, as shown in Figures 3 and 4 (Supporting Information). To evaluate the nitrogen reduction to ammonia performance, we applied three constant potentials from 1.38 to 0.58 V versus RHE and allowed the reaction to proceed 2 h at 1 sunlight intensity. As shown in Figure 3b, the corresponding current densities increased to −3.0 mA cm^−2^ with a low bias potential in the chronoamperometry tests. The disturbance was caused by the hydrogen bubbles generated on the Au catalyst surface. The ammonia product was collected from the cathode electrolyte and estimated by the indophenol blue method (Figure S5, Supporting Information).^[^ ^28^, ^29^ ^]^ As shown in Figure 3c, the ammonia production rate reached 8.3 µg cm^−2^ h at 0.98 V versus RHE. The corresponding Faradaic efficiency was 2.1% due to the high current density, and the HER occurred predominantly at the photoanode. Using Ar as the gas source, only 0.7 µg of ammonia was formed after 2 h of reaction at 0.98 V versus RHE, confirming ammonia generation via the nitrogen reduction reaction. The H_2_ products at various bias potentials were quantified by GC and the corresponding H_2_ generation rates and Faradaic efficiencies were displayed in Figure S6 (Supporting Information). The H_2_ generation rate reached highest of 350.1 µg cm^−2^ h at 0.58 V versus RHE with the Faradaic efficiency of 98.0%. N_2_H_4_, a possible by‐product, was detected by the Watt and Chrisp method (Figure S7, Supporting Information, detection wavelength: 455 nm), whereas no N_2_H_4_ was detected by UV–Vis spectrophotometry (Figure S9, Supporting Information). It has been demonstrated that nitrogen reduction into ammonia on Au surface followed the alternating pathway of associative mechanism.^[^ ^30^ ^]^ As revealed by the X‐ray powder diffraction (XRD) characterization (Figure S10, Supporting Information), the exposed surface facet of Au catalyst of the PEC device was (100) facet. The potential‐limiting step on the Au(100) surface was the protonation of N_2_ to N_2_H_2_* with the maximum free‐energy change.^[^ ^31^ ^]^ After this key step, the subsequent elementary reactions to yield ammonia are exothermic and spontaneous with the assistance of protons and electrons. The detailed NRR pathways on Au surface were displayed in Figure S11 (Supporting Information). To further confirm the source of nitrogen for ammonia generation, the resulting electrolyte was examined by NMR spectroscopy using ^14^N_2_ as the feeding gas (Figure 3d). Three hydrogen peaks were detected at 6.87, 6.96, and 7.04 ppm, corresponding to ^14^NH_3_. In addition, the isotope labelling experiment using ^15^N_2_ as the gas source for nitrogen reduction was conducted at 0.98 V versus RHE for 12 h. Two hydrogen peaks at 6.89 and 7.01 ppm were detected in the NMR spectrum, further confirmed that the ammonia in the electrolyte was generated from ^15^N_2_.^[^ ^6^, ^7^, ^8^, ^9^, ^10^, ^32^, ^33^ ^]^

As the Au/Ti/3J InGaP/GaAs/Ge cell produced 2.37 V under light illumination, a detailed PV characterizations of Au/Ti/3J InGaP/GaAs/Ge cell at various light intensities were evaluated in the air, as displayed in Figure S12 and Table S1 (Supporting Information). The power conversion efficiency (PCE) at 0.2 sunlight intensity was 17.98% and reached highest at 3 sunlight of 20.72%, revealing the highly efficient and stable solar‐to‐electricity conversion of the Au/Ti/3J InGaP/GaAs/Ge cell at various light intensities. An unassisted two‐electrode PEC measurement (Figure 1b, black circuit) was conducted to estimate the NRR performance using the PEC cell as photoanode and Pt as the counter electrode in an H‐type cell with a bipolar membrane to separate the anode from the cathode. The LSV curves in **Figure** **4**a show that the current densities increased with increasing sunlight intensities, reaching −14.9 mA cm^−2^ at 5 sunlight intensity without any bias potential. The chronoamperometry curves of the unassisted PEC NRR conducted for 2 h at various intensities are shown in Figure 4b. The photocurrent reached −2.8 mA cm^−2^ at 3 sunlight intensity. The corresponding ammonia production rates and Faradaic efficiency are shown in Figure 4c,d, respectively. The highest ammonia production rate reached 12.4 µg cm^−2^ h at 1.5 sunlight intensity, with a Faradaic efficiency of 3.1%. The Faradaic efficiency increased up to 28.9% at 0.2 sunlight intensity. The limited number of photoelectrons at a low light intensity were largely consumed by the NRR, leading to the high NH_3_ Faradaic efficiency.^[^ ^10^ ^]^ The STA efficiency was derived from the ammonia production rate and input light intensity, which was calculated as^[^ ^11^ ^]^

(1) $$\begin{array}{ccc} & & \mathrm{STA}\;\mathrm{efficiency}\left(\%\right)\\ & & \;=\frac{\begin{array}{c} \left[\Delta G^{\circ }\;{\mathrm{for}\;\mathrm{NH}}_{3}\;\mathrm{generation}\left({J\;\mathrm{mol}}^{-1}\right)\right]\\ \;\times \left[{\mathrm{NH}}_{3}\;\mathrm{formation}\left(\mathrm{mol}\right)\right] \end{array}}{\begin{array}{c} \left[\mathrm{total}\;\mathrm{input}\;\mathrm{energy}\left(W\right)\right]\\ \;\times \left[\mathrm{reaction}\;\mathrm{time}\left(s\right)\right] \end{array}}\times 100\left(\%\right) \end{array}$$

Here, the free energy *∆G°* for ammonia generation is 339 kJ mol^−1^. As shown in Figure 4e, the highest STA efficiency was achieved at 0.2 sunlight intensity (1.11%), and the efficiency decreased at higher light intensities, suggesting that the solar energy is directly and efficiently converted into ammonia at low light intensities in this unassisted PEC system. With intensive light illumination, the 3J InGaP/GaAs/Ge cell could provide a large number of electrons for competitive hydrogen generation, blocking the intrinsic NRR activity of the Au catalyst and lowering the PEC NRR performance. The ammonia selectivity was highest of 9.24% at 0.2 sunlight and decreased to 0.33% at 3 sunlight. The H_2_ generation rates and corresponding solar‐to‐hydrogen (STH) efficiencies of the two‐electrode unassisted PEC system at various sunlight intensities were shown in Figure S13 (Supporting Information). The H_2_ generation rates increased with light intensities and achieved highest of 121.7 µg cm^−2^ h at 3 sunlight intensity. In comparison, the STH efficiency achieved highest at 1.5 sunlight of 6.66% and lowest at 0.5 sunlight of 2.26%, respectively. In wired unassisted PEC NRR systems, it is difficult to achieve high nitrogen reduction current and ammonia yield due to the limited solar conversion efficiency and catalytic area. The highest ammonia production rate achieved thus far is just 1.33 µg cm^−2^ h, which is too low for practical applications. The ammonia production rate with our unassisted two‐electrode PEC system using the Au/Ti/3J InGaP/GaAs/Ge cell as photoanode (12.4 µg cm^−2^ h) is the highest achieved to date (Figure 4f).^[^ ^12^, ^13^, ^14^ ^]^

**Figure 5:**
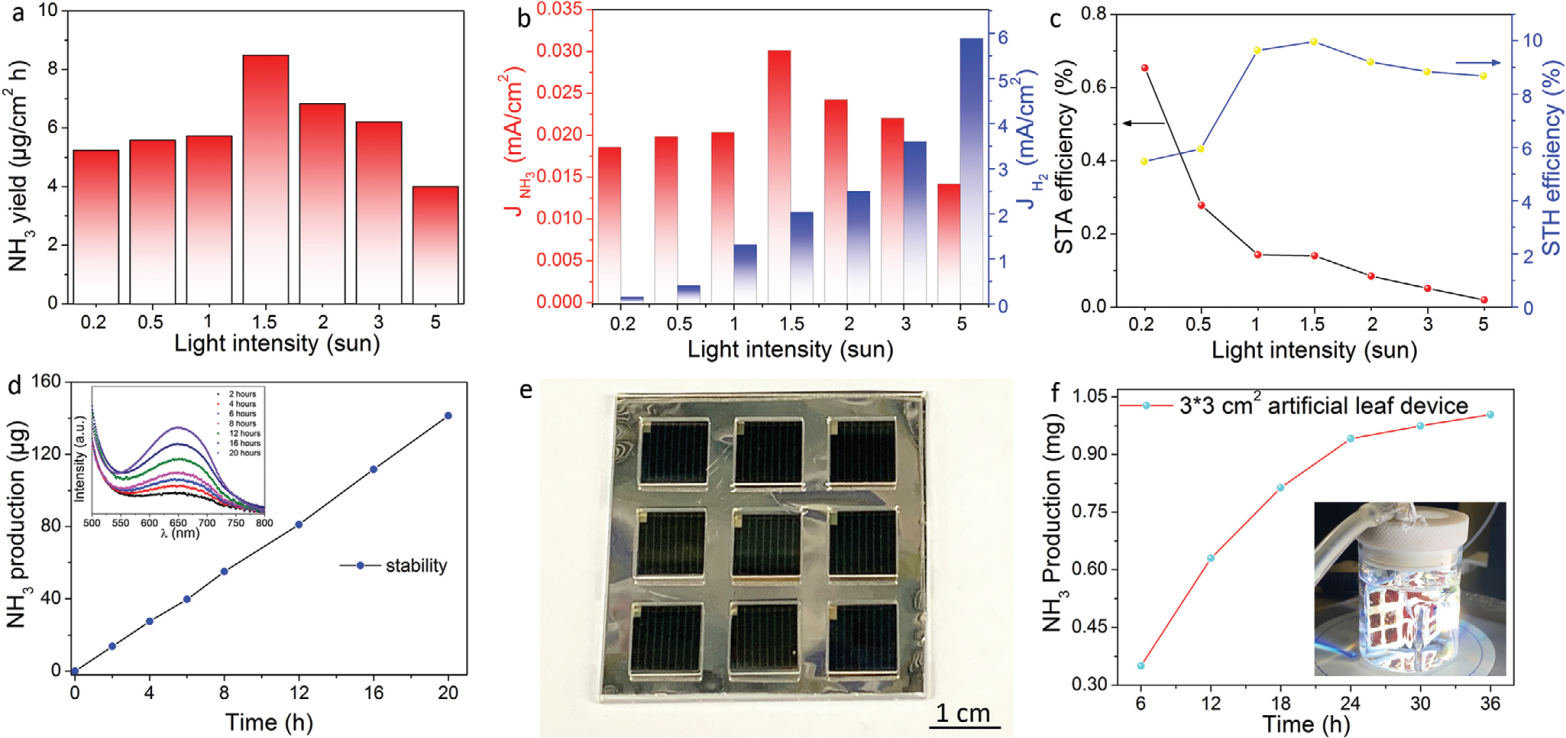
Nitrogen reduction reaction (NRR) performance of the Au/Ti/3J InGaP/GaAs/Ge/Ni foil as an artificial leaf. a) Ammonia production rates of the standalone Au/Ti/3J InGaP/GaAs/Ge/Ni foil artificial leaf under various light intensities and b) the corresponding current density of ammonia production (left column) and hydrogen production (right column). c) Solar‐to‐ammonia (STA) efficiency (left column) and solar‐to‐hydrogen (STH) efficiency (right column) under various light intensities. d) Ammonia production of the artificial leaf NRR under 1.5 sunlight illumination at 2, 4, 6, 8, 12, 16, and 20 h. Insert in (d) shows the corresponding ammonia detection by indophenol blue method. e) Photograph of the 3 × 3 cm Au/Ti/3J InGaP/GaAs/Ge/Ni foil artificial leaf before the photoelectrochemical (PEC) test. f) Ammonia production yield at 6, 12, 18, 24, 30, and 36 h by the 3 × 3 cm artificial leaf at 1.5 sunlight intensity. Insert in (f) shows the artificial leaf in real working condition. Reaction conditions: N_2_‐saturated 0.1 m NaOH electrolyte under various sunlight illumination (a–c) and 1.5 sunlight illumination (d,f).

Simulating the natural photosynthesis, an artificial leaf can directly convert solar energy into chemical energy to produce valuable chemicals. By integrating a Ni foil for the OER, we fabricated a monolithic artificial leaf with the Au/Ti/3J InGaP/GaAs/Ge/Ni foil cell. The PV performance was evaluated under ambient conditions at 1 sunlight intensity, as shown in Figure S14 (Supporting Information). *V*_oc_ was maintained at 2.37 V, while J_sc_ dropped to 9.1 mA cm^−2^. The FF decreased to 73.90% due to the unsatisfactory contact between the Au/Ti/3J InGaP/GaAs/Ge cell and the Ni foil. The nitrogen fixation performance was evaluated in 0.1 m NaOH at various light intensities, as shown in **Figure** **5**a. At 0.2 sunlight intensity, an ammonia production rate of 5.1 µg cm^−2^ h was achieved using the wireless device. Upon increasing the intensity to 1.5 sunlight, the artificial leaf device exhibited the highest ammonia production rate of 8.5 µg cm^−2^ h. The hydrogen production rates were also evaluated in the continuous chronoamperometry test using GC, as shown in Figure S15 (Supporting Information). The hydrogen yield increased with increasing light intensity and reached 175.6 µmol h^−1^ at 5 sunlight intensity. The corresponding photocurrent for ammonia and hydrogen production is shown in Figure 5b. *J*_NH3_ was the highest (0.031 mA cm^−2^) at 1.5 sunlight intensity and decreased to 0.015 mA cm^−2^ at 5 sunlight intensity. In contrast, *J*_H2_ increased with increasing sunlight intensity and reached 5.9 mA cm^−2^ at 5 sunlight intensity. The STA efficiency of the artificial leaf (derived from the production rate and light intensity) reached 0.68% at 0.2 sunlight intensity and decreased to 0.02% at 5 sunlight intensity (Figure 5c, left column). At the same time, we also evaluated the STH efficiency (Figure 5c, right column). From 0.2 to 1.5 sunlight intensity the STH efficiency increased up to 10%. At low light intensities, the 3J InGaP/GaAs/Ge cell absorbed limited photons to generate electrons and holes. A large proportion of electrons were consumed to reduce adsorbed nitrogen into ammonia by the Au catalyst, resulting in high STA efficiency. However, under intensive light illumination, the PEC device provided ample electrons for the HER. The rapidly hydrogen generation occupied the catalytic Au sites for the NRR, thereby decreasing the STA efficiency. Increasing the intensity to 5 sunlight increased the surrounding temperature and generated large amount of hydrogen bubbles on the photoelectrode surface, which further hindered the light absorption and decreased the STA and STH efficiencies. It is worth noting that the STA efficiency of the PEC artificial leaf decreased to half that of the unassisted wired two‐electrode PEC system, owing to the poor charge carrier transport in the electrolyte and the poor conductivity of the surface protection layer.^[^ ^9^ ^]^ To reveal the important role of Au catalyst in NRR, we deposited Pt and Cu with 20 nm in thickness onto the Ti/3J InGaP/GaAs/Ge cell. The PV performances were displayed in Figure S16a (Supporting Information). The *V*_oc_ and *J*_sc_ of Pt‐based PEC cell was 2.37 V and 8.99 mA cm^−2^. In addition, the *V*_oc_ and *J*_sc_ of Cu‐based PEC cell was 1.82 V and 2.96 mA cm^−2^, which was much lower than the Au‐based one. We integrated the Pt and Cu‐based PEC cell with Ni foil and conducted the NRR at 1.5 sunlight intensity. Only H_2_ was detected by GC and no ammonia/N_2_H_4_ was detected by UV–Vis absorption analysis. The H_2_ production rate of the Pt and Cu‐based artificial leaves were lower than the Au‐based one, as shown in Figure S16b (Supporting Information). We further determined the stability of the artificial leaf at 1.5 sunlight intensity. The ammonia production increased linearly up to 141.0 µg in the 20‐h stability test, as shown in Figure 5d. The results of the corresponding time‐dependent UV–Vis absorption analysis by the indophenol blue method are shown in the insert of Figure 5d, which clearly demonstrate the ammonia generation by our artificial leaf. To confirm the ammonia production from the N_2_ reduction, we performed the artificial leaf nitrogen fixation using ^15^N_2_ gas as nitrogen source. A series of ^15^NH_4_^+^ solution were prepared and characterized by NMR, as shown in Figure S8 (Supporting Information). The signal intensities at 6.89 and 7.01 ppm were linearly increased with the ^15^NH_4_^+^ concentration. The artificial leaf nitrogen fixation was conducted at 1.5 sunlight intensity for 2 hours using ^15^N_2_ as the feeding gas. The electrolyte was collected and characterized by NMR, as shown in Figure S17a (Supporting Information). The ammonia production rate was 8.2 µg cm^−2^ h, consisted with the ammonia production rate of 8.5 µg cm^−2^ h at the same condition using ^14^N_2_ as the feeding gas (Figure S17b, Supporting Information).

Since the artificial leaf using the 3J InGaP/GaAs/Ge cell is wireless, it can be implemented at any level in a highly distributed manner. To further confirm the excellent PEC performance and achieve the ammonia production at the mg‐scale, we enlarged the artificial leaf by integrating nine pieces of 1 × 1 cm Au/Ti/3J InGaP/GaAs/Ge cells on a 3 × 3 cm Ni foil, as shown in Figure 5e. The ammonia generation was measured at 1.5 sunlight intensity for 36 h in the 0.1 m NaOH electrolyte solution (Figure 5f). As shown in Figure 5f, the enlarged artificial leaf produced 1.0039 mg of ammonia after 36‐h of light illumination. The photograph of the 3 × 3 cm artificial leaf after the PEC test is shown in Figure S18 (Supporting Information). The wireless unassisted PEC system integrated the light absorption components with catalytically active sites, achieving high solar‐driven ammonia conversion efficiency and high ammonia yield simultaneously.

## 3 Conclusion

Ammonia is emerging as an important candidate as a renewable energy carrier. Using 3J InGaP/GaAs/Ge/Ni, we have demonstrated for the first time a truly wireless artificial leaf that can achieve 0.68% STA efficiency at 0.2 sunlight intensity and 8.5 µg cm^−2^ h ammonia production rate at 1.5 sunlight intensity. A 3 × 3 cm artificial leaf produced 1.0039 mg of ammonia in the 36‐h illumination test. Our work sets a roadmap for navigating the pathway toward the establishment of large‐scale technologies for solar‐driven ammonia conversion at the industrial level.

## 4 Experimental Section

### Fabrication of the Au/Ti/3J InGaP/GaAs/Ge/Ni foil PEC Cell

To fabricate the Au/Ti/3J InGaP/GaAs/Ge/Ni foil artificial leaf, a protective 10 nm thick Ti layer was deposited on the InGaP side of the cell by sputtering Ti metal (99.9%). Then, a 20 nm thick Au catalyst layer was deposited above the Ti layer using the e‐beam evaporation technique at a deposition rate of 0.4A s^−1^ and high vacuum pressure of 1 × 10^−07^ Torr. On the reverse side, a Ga‐In eutectic alloy (Sigma–Aldrich) was deposited on the Ge side of the cell to make ensure ohmic contact with the Ni foil (1 × 1 cm). The device was subsequently connected with a Cu wire using silver paste and then embedded in Epoxy (Hysol 11C). The only part of the reverse side was covered with the Ni foil as the OER catalyst and exposed to the electrolyte. Finally the Epoxy was dried at 80 °C for 30 min before use. In a similar manner, the Au/Ti/3J InGaP/GaAs/Ge photoanode was fabricated by replacing the Ni foil with a quartz sheet (1 × 1 cm). The Pt and Cu‐based PEC devices were prepared in the same way using the e‐beam evaporation technique and Pt/Cu sources.

### Photoelectrochemical N_2_ Reduction Using the Au/Ti/3J InGaP/GaAs/Ge Cell

The PEC measurements were carried out in a gas‐tight H‐type electrolytic cell using three (two)‐electrode or wireless systems without iR compensation. The electrolysis tests were performed on an electrochemical workstation (BioLogic VMP300). During the PEC measurements, various light intensities were obtained using a 150 W halogen‐lamp‐based solar simulator (ABET technologies Sun 2000) equipped with a PMMA (Edmund Optics #43‐025) Fresnel lens. Chronoamperometry measurements were carried out using three (two)‐electrode systems in the 0.1 m NaOH electrolyte solution. The electrolyte was stirred at 400 rounds per minute throughout the test. N_2_ gas was purged into the cathodic chamber at a fixed flow rate of 10 SCCM. After at least 30 min to remove air, controlled PEC experiments were performed at applied bias potentials and light intensities for 2 h. The cathodic electrolytes were collected for further analysis. For the artificial leaf NRR, the integrated PEC device was put into an air‐tight quartz cell with N_2_ saturated 0.1 m NaOH electrolyte. Light intensities were controlled by changing the distance from the solar simulator. Gas products was analyzed using the online gas chromatograph during the PEC nitrogen fixation. After 2 h light illumination, the electrolytes were collected for further analysis.

### Quantification of Ammonia

The concentration of ammonia product was determined by the indophenol blue method. Briefly, 2 mL of 1 m NaOH solution containing salicylic acid (5 wt%) and sodium citrate (5 wt%) was mixed with 2 mL of the cathodic electrolyte after the PEC experiment. Then 1 mL of 0.05 m NaClO and 0.2 mL of C_5_FeN_6_Na_2_O (1 wt%) were added into the previous solution. The absorption spectrum was recorded using an UV–Vis absorption spectrophotometer (UV‐Vis‐NIR‐Lambda 950_acl). The concentration of ammonia was determined from the absorbance at 655 nm. The concentration versus absorbance curve was calibrated using a series of standard ammonium chloride solutions of different concentrations.

### Quantification of Hydrazine

The concentration of hydrazine product was determined by the Watt and Chrisp method. Briefly, a mixture of para‐(dimethylamino) benzaldehyde (0.599 g), HCl (12 mol L^−1^, 3 mL), and ethanol (30 mL) was used as a color reagent. An amount of 2 mL of the electrolyte after electrolysis was mixed with 2 mL of color reagent. The absorption spectrum was recorded using an UV–Vis absorption spectrophotometer (UV‐Vis‐NIR‐Lambda 950_acl). The concentration of hydrazine was determined from the absorbance at 455 nm. The concentration versus absorbance curve was calibrated using standard hydrazine hydrate solutions of different concentrations.

### Calculation of Faradaic Efficiency

The Faradaic efficiency for ammonia production was calculated at a controlled potential as follows:

(2) $$FE=\left[C_{NH3}\times V\times N\times F\right]/Q$$

FE: Faradaic Efficiency
*C*_NH3_: measured ammonia concentration
*V*: volume of cathodic NaOH electrolyte
*N*: number of electrons transferred for product formation (3 for ammonia)
*F*: Faraday constant (96 485 C mol^−1^)
*Q*: quantity of electric charge integrated by the *i‐t* curve.

### Calculation of Solar‐to‐Hydrogen (STH) Efficiency

During the PEC nitrogen fixation, hydrogen was generated as a result of the competitive HER. The quantity of hydrogen was detected by an online gas chromatograph (Agilent 7890B) at various light intensities. The STH efficiency was calculated as follows:

(3) $$\begin{array}{ccc} & & \mathrm{STH}\;\mathrm{efficiency}\left(\%\right)\\ & & \;=\frac{\left[\Delta G^{\circ }\;{\mathrm{for}\;H}_{2}\;\mathrm{generation}\;({J\;\mathrm{mol}}^{-1})]\times [H_{2}\;\mathrm{formation}\left(\mathrm{mol}\right)\right]}{\left[\mathrm{Total}\;\mathrm{input}\;\mathrm{energy}\left(W\right)\right]\times \left[\mathrm{Reaction}\;\mathrm{time}\left(s\right)\right]}\\ & & \;\;\;\;\times \;100\left(\%\right) \end{array}$$

Here, the free energy for hydrogen generation is 237 kJ mol^−1^.

### Characterization

The Au/Ti/3J InGaP/GaAs/Ge cell was prepared using the focused ion beam (Helios 400 S, FEI) technique before the TEM test. To protect the sample from ion beam damage, a Pt layer (300 nm) was deposited using electron beam on the InGaP side. HAADF–STEM imaging and energy dispersive spectroscopy mapping were performed on a transmission electron microscope (Titan Themis Z, FEI) equipped with a high‐brightness electron gun (x‐FEG), an electron beam monochromator and a double Cs corrector operated at 300 kV. The EQE spectra were obtained using a spectral response system (Enli Technology Co., Ltd. R3011). The absorbance, reflectance, and transmittance spectra of the 3J InGaP/GaAs/Ge cell were measured from the InGaP side in the air using the UV–Vis spectrophotometer (UV‐Vis‐NIR‐Lambda 950_acl). For the indophenol blue and Watt and Chrisp method, the absorption spectrum was obtained on a UV–Vis spectrophotometer (UV‐Vis‐NIR‐Lambda 950_acl). XRD pattern was obtained at room temperature by a Bruker D2 PHASER powder diffractometer (German Bruker) equipped with a LynxEye detector and a Cu source. The ^1^H NMR spectra were recorded using a Bruker 600 MHz NMR instrument. The hydrogen product and purified N_2_ gas were quantified by an online gas chromatograph (Agilent 7890B) equipped with a Shincarbon column, a thermal conductivity detector (TCD), and a flame ionization detector (FID) with a methanizer. Ultrahigh‐purity Ar (99.999%) was used as the carrier gas. The gas samples were detected using an air‐tight syringe every 5 min to determine the H_2_ concentration and N_2_ purity.

## Conflict of Interest

The authors declare no conflict of interest.

## Supporting information

Supporting Information

## Data Availability

Research data are not shared.
