# Supplementary material for: Artificial Leaf for Solar‐Driven Ammonia Conversion at Milligram‐Scale Using Triple Junction III‐V Photoelectrode
Source: Adv Sci (Weinh). 2023 Mar 22;10(14):2205808. doi: 10.1002/advs.202205808 (PMC10190632; doi:10.1002/advs.202205808)
Supplement: Supplementary file 1 — Supporting Information [file ADVS-10-2205808-s001.pdf]

ADVANCED SCIENCE
Open Access

Supporting Information

for *Adv. Sci.*, DOI 10.1002/advs.202205808

Artificial Leaf for Solar-Driven Ammonia Conversion at Milligram-Scale Using Triple Junction III-V Photoelectrode

Hao Huang, Dharmaraj Periyanagounder, Cailing Chen, Zhongxiao Li, Qiong Lei, Yu Han, Kuo-Wei Huang and Jr-Hau He**

Supplementary Information

An Artificial Leaf for Solar-driven Ammonia Conversion at Milligram-scale Using Triple Junction III-V Photoelectrode

Hao Huang,[1,2] Dharmaraj Periyanagounder,[1] Cailing Chen,[2,3] Zhongxiao Li,[4] Qiong Lei,[2,3] Yu Han,[2,3] Kuo-Wei Huang[1,2*] and Jr-Hau He[4*]

[1]*KAUST Catalysis Center, King Abdullah University of Science and Technology, Thuwal, 23955-6900, Saudi Arabia.*

[2]*Division of Physical Sciences and Engineering, King Abdullah University of Science and Technology, Thuwal, 23955-6900, Saudi Arabia.*

[3]*Advanced Membrances and Porous Materials Center, King Abdullah University of Science and Technology, Thuwal, 23955-6900, Saudi Arabia.*

[4]*Department of Materials Science and Engineering, City University of Hong Kong, Kowloon, Hong Kong SAR 999077, China.*

[*]Corresponding author: *jrhauhe@cityu.edu.hk; kuowei.huang@kaust.edu.sa*

Chemicals. Hydrochloric acid (HCl, 12 mol/L), sulfuric acid (H_2SO_4, 96-98%), sodium hydroxide ($NaOH$), ammonium chloride (NH_4Cl), ^{15}N labeled ammonium chloride ($^{15}NH_4Cl$), hydrazine hydrate ($N_2H_4 \cdot H_2O$), sodium hypochlorite solution ($NaClO$, 5.2% w/v), salicylic acid ($C_7H_6O_3$), trisodium citrate dehydrate ($C_6H_5Na_3O_7 \cdot 2H_2O$), sodium nitroferricyanide dehydrate ($C_5FeN_6Na_2O \cdot 2H_2O$), methanol, ethanol, Nafion solution and bipolar exchange membrane were purchased from Sigma-Aldrich. Deionized (DI) water, with a resistivity of 18.2 MΩ·cm, was produced from Millipore Milli-Q grade. All chemicals were used without further purification.

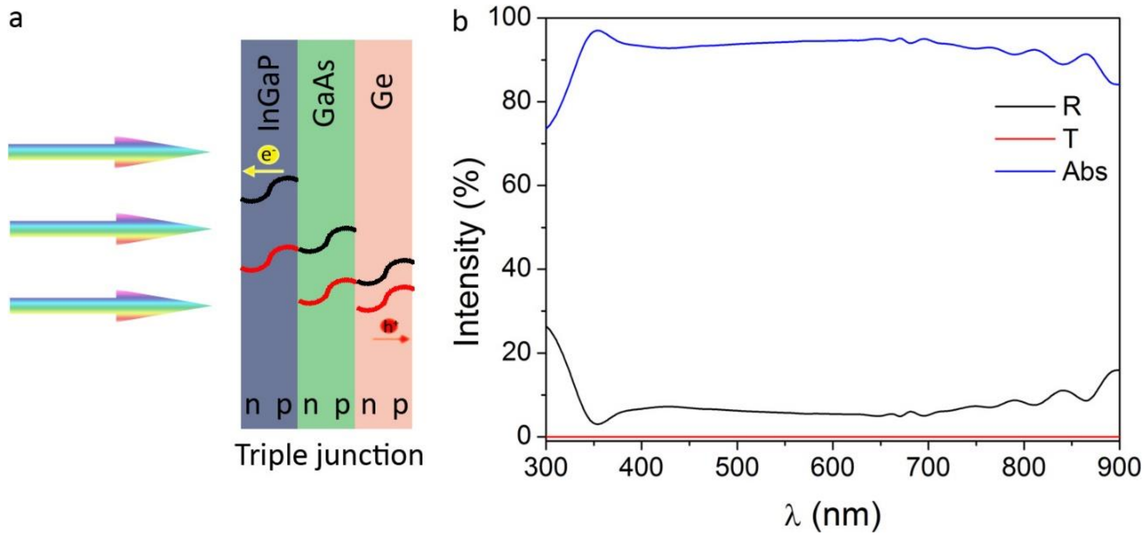

Supplementary Figure 1 | (a) Cascade band structure of the 3J InGaP/GaAs/Ge cell. **(b)** Absorbance (Abs), reflectance (R) and transmittance (T) spectra of the 3J InGaP/GaAs/Ge cell measured from the InGaP surface in the air.

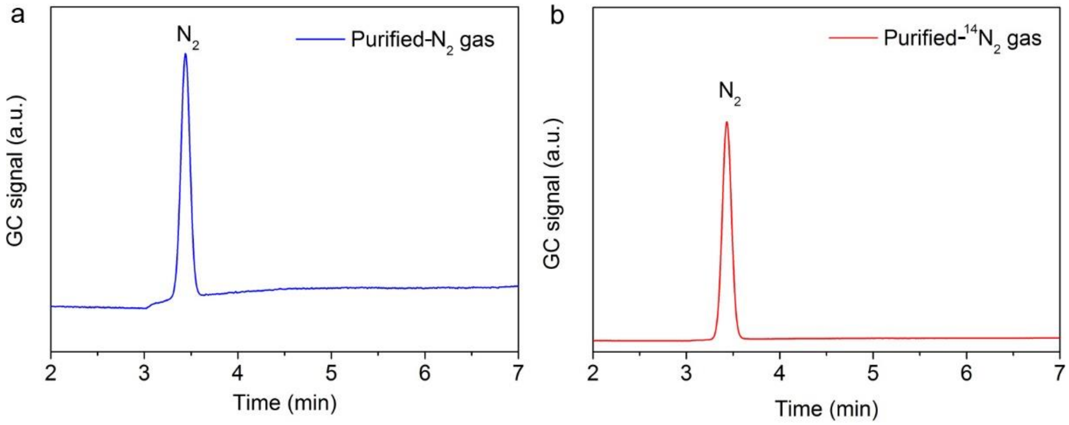

Supplementary Figure 2 | Gas chromatography spectra of **(a)** purified-N_2 and **(b)** purified-$^{14}N_2$ gas. Only the N_2 signal was detected, indicating the purified N_2 gas used in this work contained no NO_x containment.

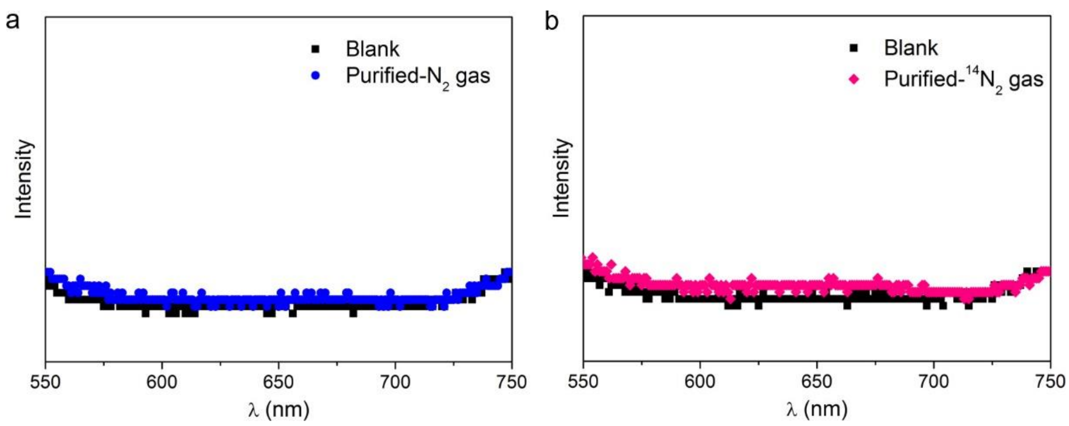

Supplementary Figure 3 | UV-vis absorption spectra for the determination of ammonia containments in **(a)** the blank (deionized water) and **(b)** 0.1 M NaOH electrolyte using purified-N_2 and $^{14}N_2$ gases.

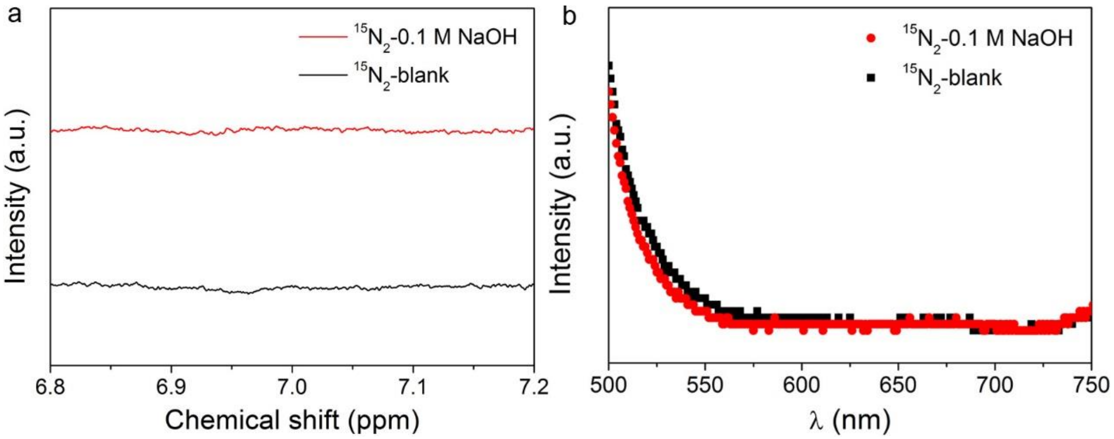

Supplementary Figure 4 | (a) NMR and **(b)** UV-vis absorption spectra for the determination of ammonia containments in the blank (deionized water) and 0.1 M NaOH electrolyte using $^{15}N_2$ gas.

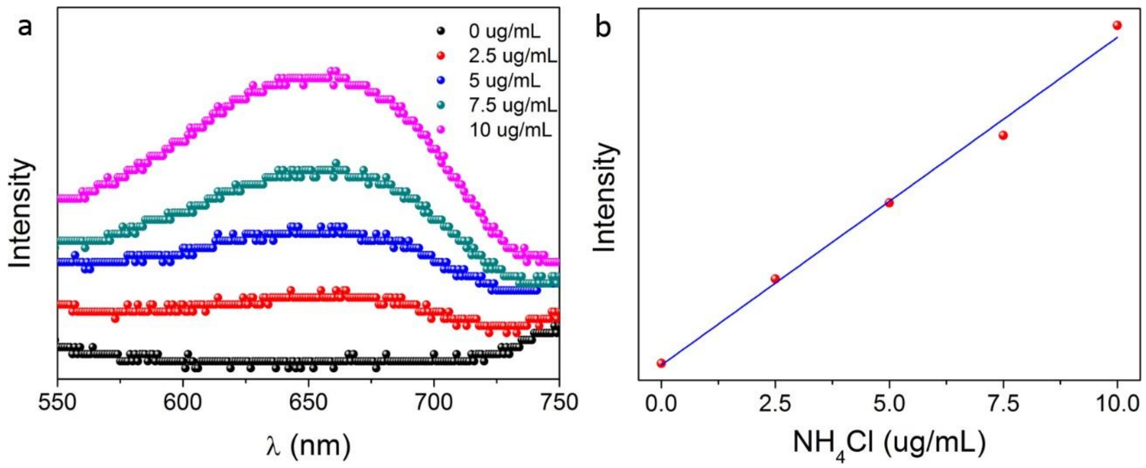

Supplementary Figure 5 | (a) UV-vis curves and **(b)** concentration-absorbance curve of NH_4^+ ions solution with a series of standard concentration. The absorbance at 655 nm was measured by UV-vis spectrophotometer. The standard curve showed good linear relation of absorbance with NH_4^+ ion concentration.

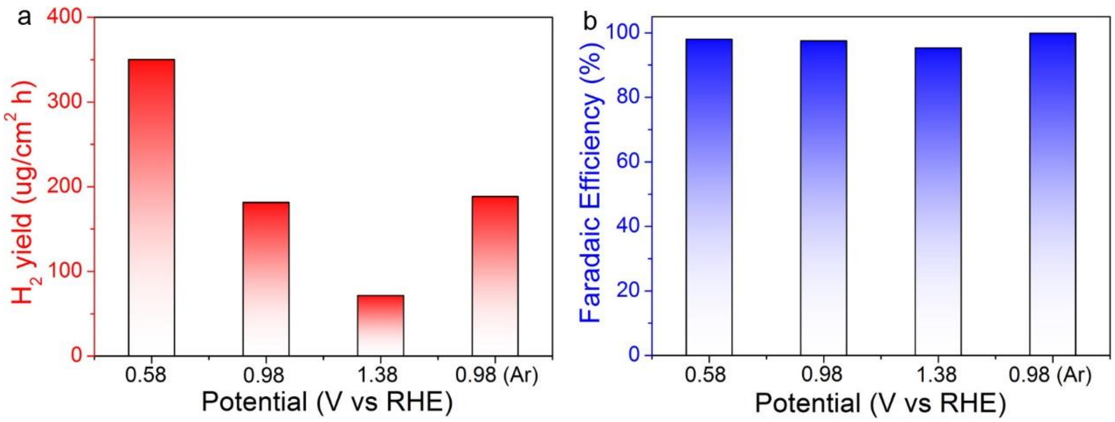

Supplementary Figure 6 | (a) H_2 generation rates and **(b)** the corresponding Faradaic Efficiency of the Au/Ti/3J InGaP/GaAs/Ge cell with various bias potential vs RHE.

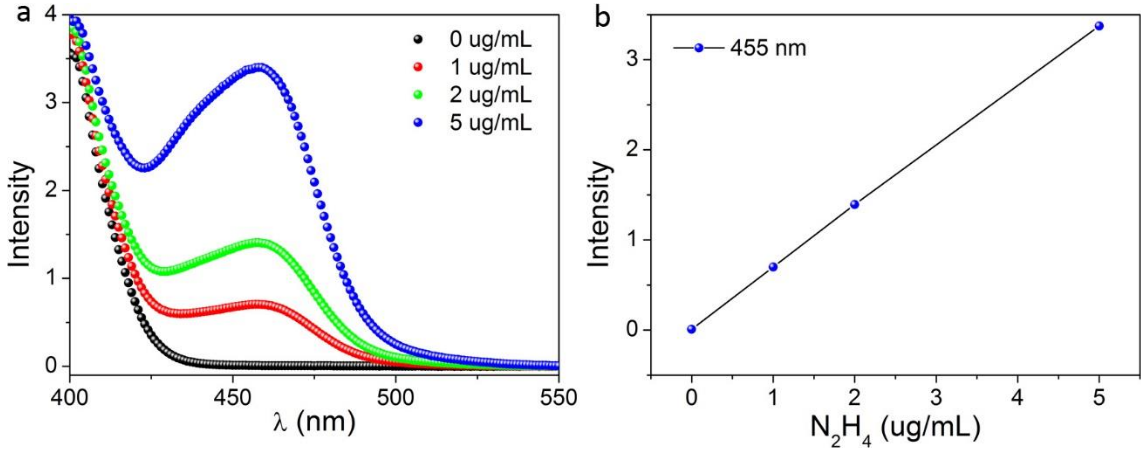

Supplementary Figure 7 | (a) UV-vis curves and **(b)** concentration-absorbance curve of N_2H_4 solution with a series of standard concentration. The absorbance at 455 nm was measured by UV-vis spectrophotometer. The standard curve showed good linear relation of absorbance with N_2H_4 concentration.

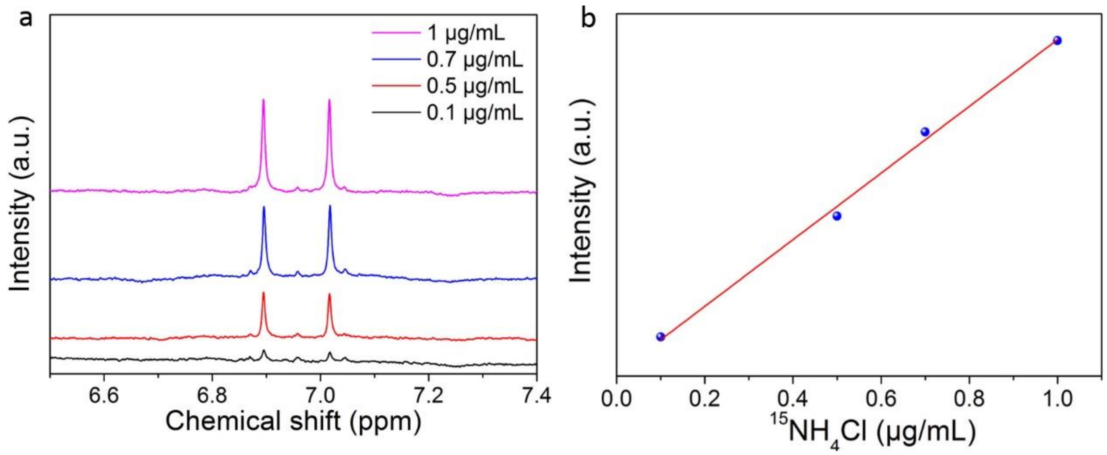

Supplementary Figure 8 | (a) NMR spectra and **(b)** concentration-intensity curve of $^{15}NH_4^+$ ions solution with a series of standard concentration. The standard curve showed good linear relation of intensity with $^{15}NH_4^+$ ion concentration.

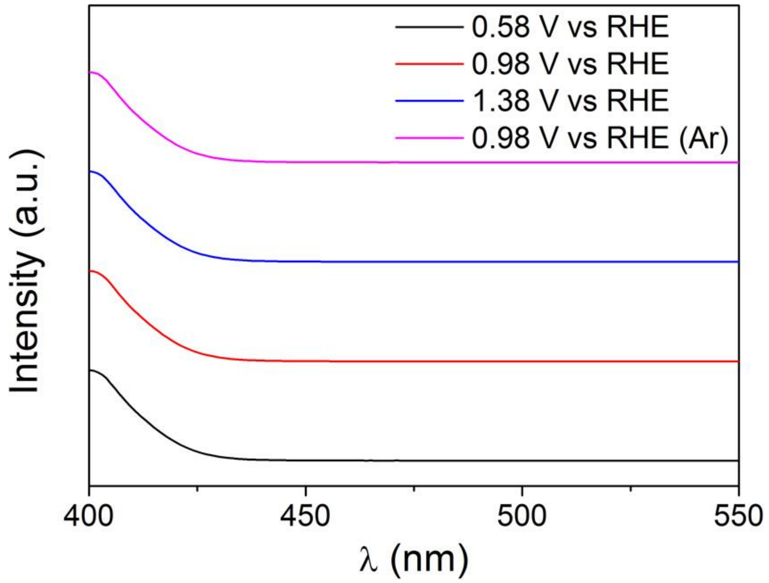

Supplementary Figure 9 | UV-vis absorption spectra of N_2H_4 detection of the Au/Ti/3J InGaP/GaAs/Ge cell with various bias potential vs RHE.

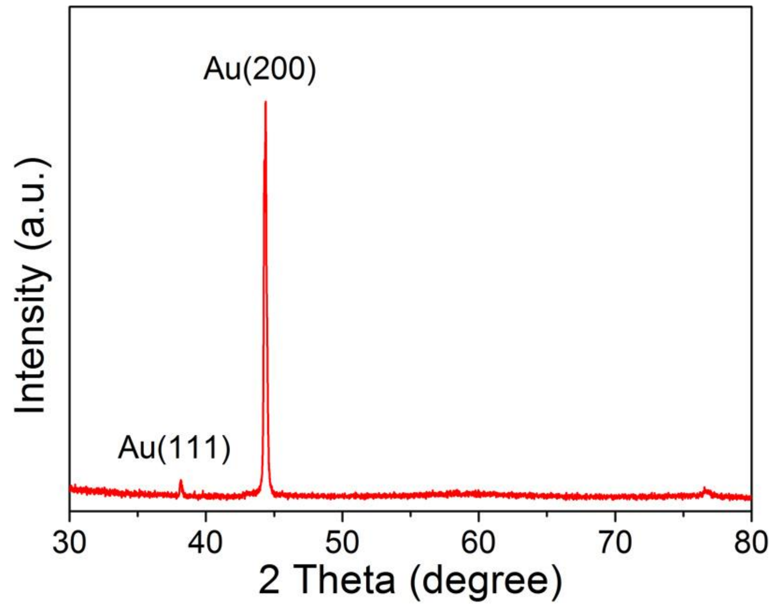

Supplementary Figure 10 | XRD pattern of the Au/Ti/3J InGaP/GaAs/Ge cell from the Au surface.

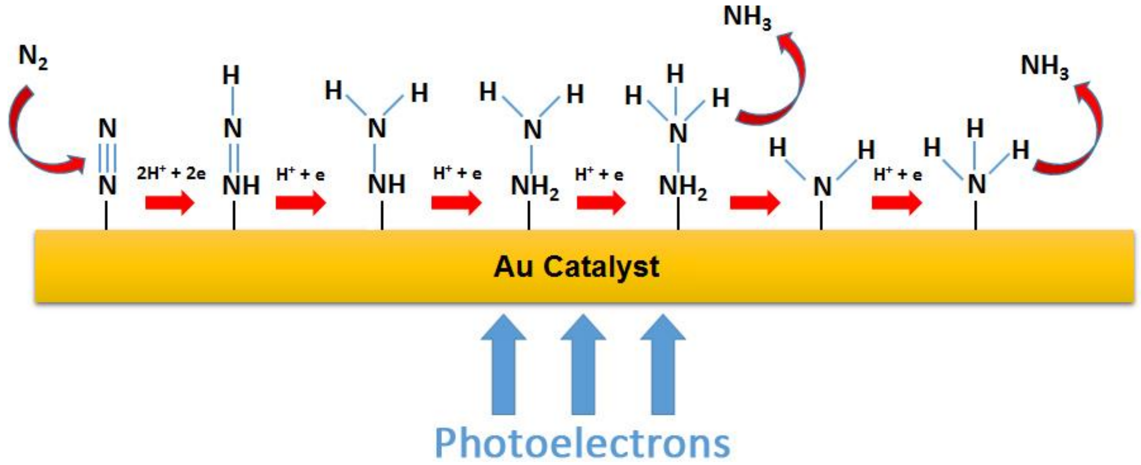

Supplementary Figure 11 | Schematic illustration of the nitrogen reduction following the alternating pathway on the Au catalyst surface with the assistance of photoelectrons.

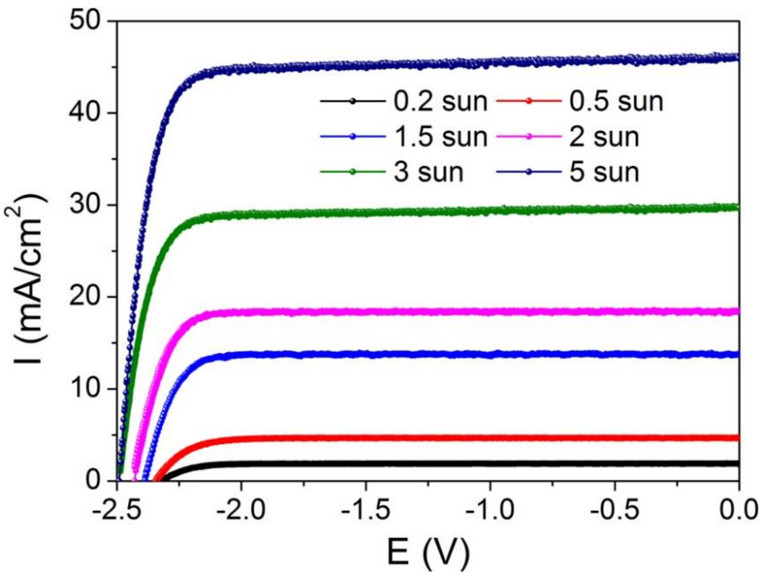

Supplementary Figure 12 | The PV characterizations of Au/Ti/3J InGaP/GaAs/Ge cell at various light intensities.

Supplementary Table 1 | The J_{sc}, V_{oc}, FF, and PCE of Au/Ti/3J InGaP/GaAs/Ge cell at various light intensities.

Number of suns	J_{sc}(mA/cm^2)	V_{oc}(V)	FF(%)	PCE(%)
0.2	1.85	2.34	83.06	17.98
0.5	4.68	2.35	82.76	18.20
1	9.26	2.37	83.18	18.25
1.5	13.89	2.38	84.09	18.53
2	18.35	2.43	85.04	18.98
3	29.82	2.49	83.72	20.72
5	46.15	2.49	83.77	19.25

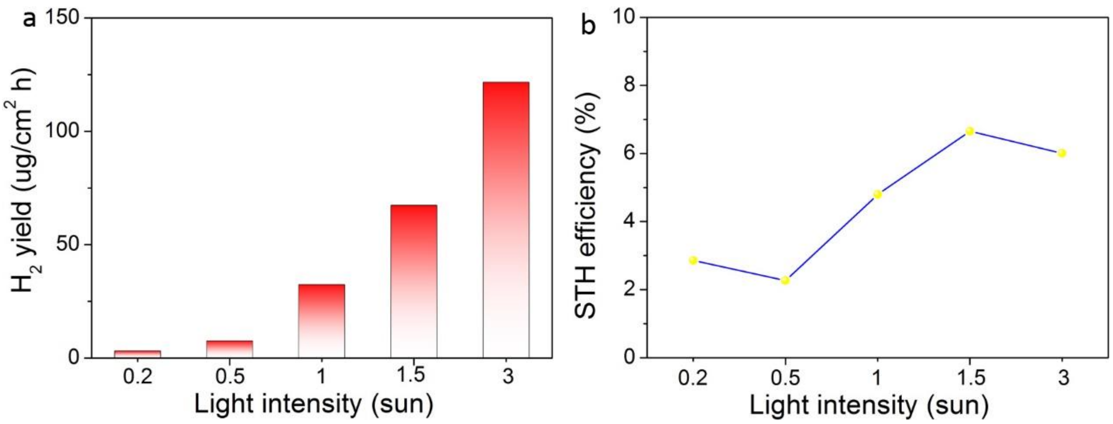

Supplementary Figure 13 | (a) H_2 production rate and **(b)** STH efficiency of the two-electrode wired PEC system under various light intensities.

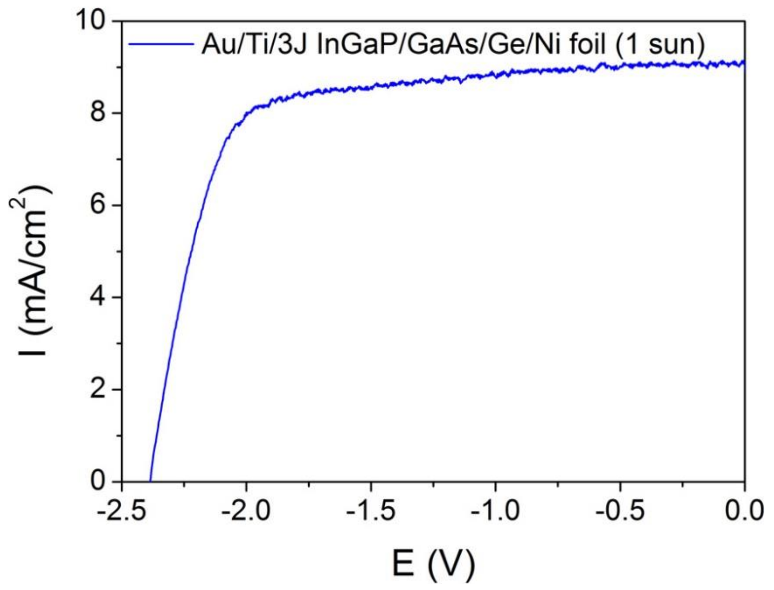

Supplementary Figure 14 | *J-V* characteristics of the Au/Ti/3J InGaP/GaAs/Ge/Ni foil under the ambient conditions under 1 sun AM 1.5G illumination.

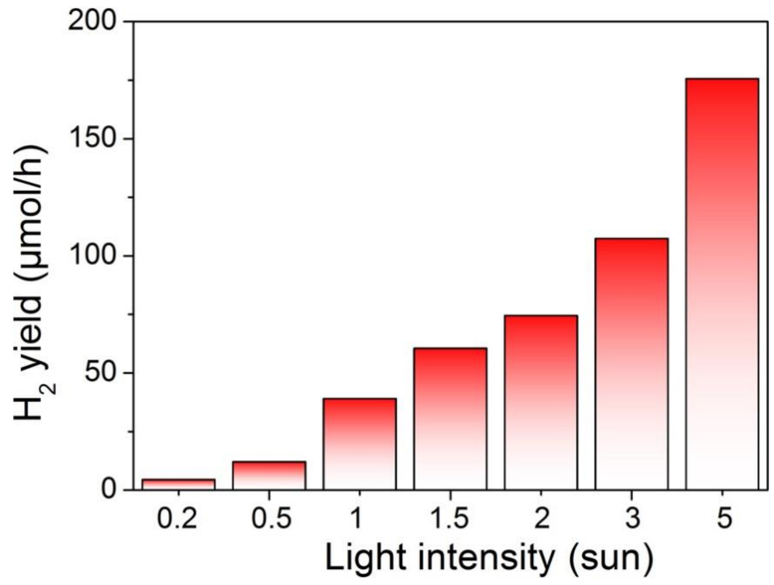

Supplementary Figure 15 | Hydrogen production rate of the monolithic Au/Ti/3J InGaP/GaAs/Ge/Ni foil artificial leaf under various light intensities.

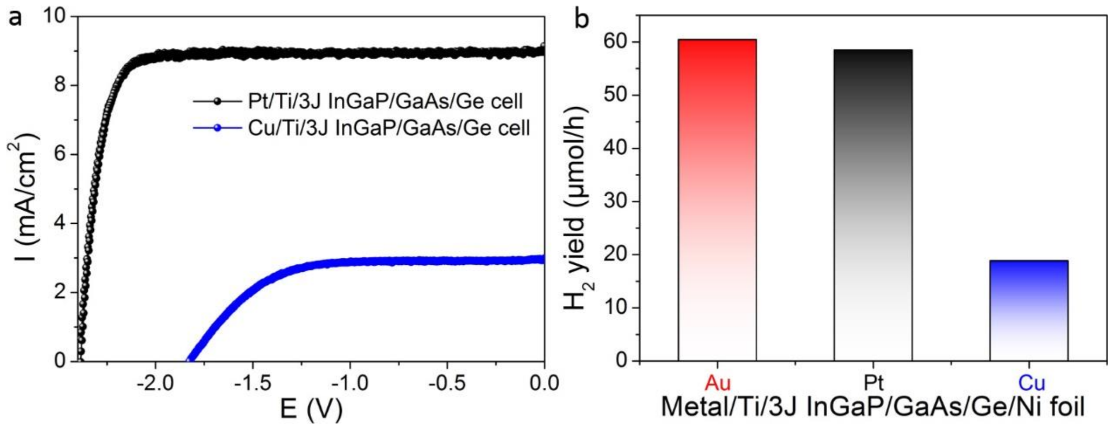

Supplementary Figure 16 | (a) J-V characteristics of the Pt/Ti/3J InGaP/GaAs/Ge cell (black) and Cu/Ti/3J InGaP/GaAs/Ge cell (blue) at ambient conditions under 1 sun AM 1.5G illumination. **(b)** Hydrogen production rate of the monolithic Metal/Ti/3J InGaP/GaAs/Ge/Ni foil artificial leaf under 1.5 sunlight intensity.

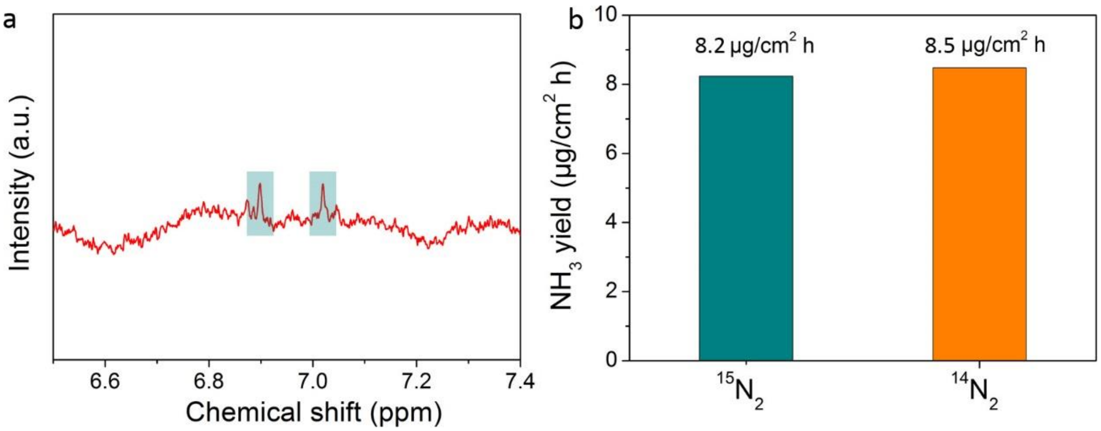

Supplementary Figure 17 | (a) NMR spectrum of the artificial leaf fixing nitrogen into ammonia at 1.5 sunlight intensity using $^{15}N_2$ as feeding gas for 2 h. **(b)** Comparison of ammonia production rates using $^{15}N_2$ and $^{14}N_2$ as feeding gases of the artificial leaf at 1.5 sunlight intensity.

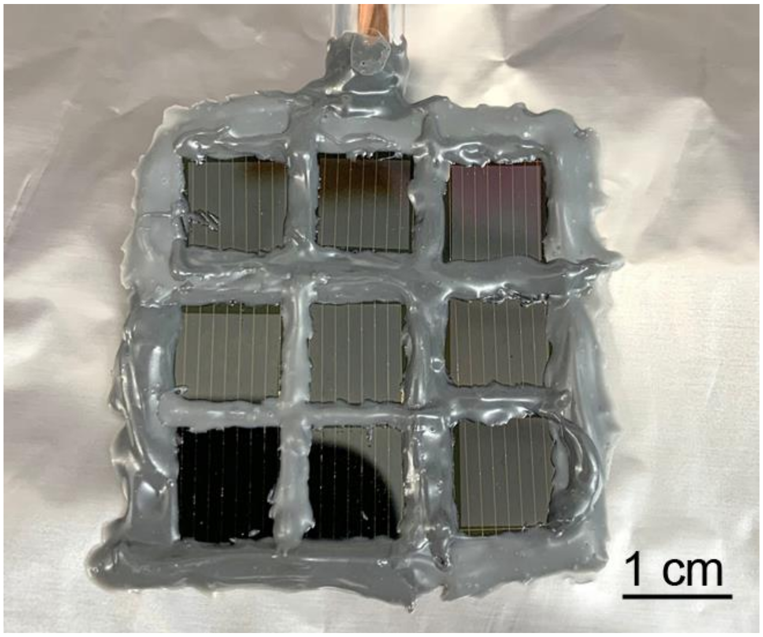

Supplementary Figure 18 | Photograph of the 3 × 3 cm Au/Ti/3J InGaP/GaAs/Ge/Ni foil artificial leaf after the 36-h PEC test.
